# Intraductal Papillary Mucinous Neoplasm of the Pancreas Arising in a Patient With Limited Cutaneous Systemic Sclerosis

**DOI:** 10.7759/cureus.15197

**Published:** 2021-05-23

**Authors:** Alex Azzam, Martin Azzam, Salman Arif

**Affiliations:** 1 Internal Medicine, University of Arizona College of Medicine - Tucson, Tucson, USA; 2 Dermatology, University of Missouri School of Medicine - Columbia, Columbia, USA; 3 Transitional Year, Southern Hills Hospital and Medical Center, Las Vegas, USA; 4 Internal Medicine, Southern Hills Hospital and Medical Center, Las Vegas, USA

**Keywords:** systemic sclerosis, scleroderma, limited cutaneous, crest syndrome, raynaud, cancer, malignancy, pancreas, intraductal papillary mucinous neoplasm

## Abstract

Systemic sclerosis (SS) is a heterogenous autoimmune disease that manifests itself with skin and internal organ involvement. The association of SS and malignancy is an emerging field of study with limited data in the literature. This report highlights the unique case of a patient with limited cutaneous SS (lcSS) found to have an intraductal papillary mucinous neoplasm (IPMN) of the pancreas. In this report, we review the clinical manifestations, serologic findings, and phenotypes of SS. Furthermore, an evaluation of the risk of pancreatic neoplasms in patients with SS will be discussed, as well as the correlation of cancers among SS phenotypes and auto-antibodies. As part of our research, a PubMed search of the following terms was performed: “systemic sclerosis, scleroderma, limited cutaneous systemic sclerosis, CREST syndrome, Raynaud syndrome, cancer, malignancy, pancreas, and intraductal papillary mucinous neoplasm".

## Introduction

Systemic sclerosis (SS) is a heterogenous autoimmune disease that is characterized by the involvement of the skin, subcutaneous tissue, and internal organs. It is a rare condition with a prevalence of approximately 30 per 100,000 people in North America and has a female predominance of about 4:1 [1]. SS is classified based on phenotype, the two major subtypes being limited cutaneous SS (lcSS) and diffuse cutaneous SS (dcSS). Another entity is SS overlap syndrome in which a patient with SS has another rheumatologic condition. SS overlap syndrome affects approximately 20% of SS patients and is most commonly associated with myositis, rheumatoid arthritis, Sjogren’s syndrome, and systemic lupus erythematosus [2]. The pathogenesis tends to be chronic and progressive and relies on three processes including vasculopathy, inflammation, and fibrosis [3]. While cancer being linked with SS is an emerging area of study, there is little data about pancreatic neoplasms in these patients.

In this report, we describe a cachectic female with lcSS who presented to the hospital with severe nausea and vomiting. She was anti-centromere antibody positive and MRI demonstrated intraductal papillary mucinous neoplasm (IPMN) and pancreatic atrophy.

## Case presentation

A 66-year-old Caucasian woman presented to the emergency department with constant, sharp abdominal pain, and intractable nausea/vomiting that had acutely worsened over the past four days. She endorsed having these symptoms intermittently for approximately one year, but never this severe. The patient indicated that she had difficulty holding down food and fluids for the past several months and was being followed by gastroenterology on an outpatient basis with an appointment upcoming. On further review of systems, the patient reported generalized weakness, fatigue, and loss of appetite. She denied fever, chills, chest pain, diarrhea, constipation, foul-smelling stool, and rash.

The patient’s past medical history was notable for esophageal candidiasis, esophageal strictures, hiatal hernia, hyperlipidemia, and hypertension. Her only relevant surgical history was a remote cholecystectomy decades ago. She had seen gastroenterology in the past but stated that an etiology for her symptoms had yet to be found. Home medications included nifedipine, pantoprazole, and ondansetron. The patient had been hospitalized once in the past year at another facility with similar symptoms, but they resolved quickly with anti-emetics and she was recommended for gastroenterology follow-up on an outpatient basis. Family history was notable for a father with multiple sclerosis. Social history was notable for 22-pack-year smoking history, occasional marijuana use, and no diet changes or recent travel. The patient denied alcohol use.

**Figure 1:**
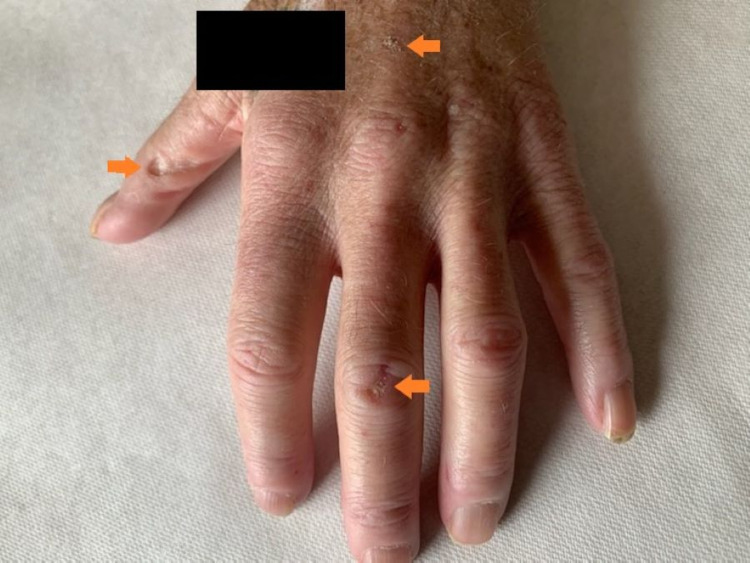
Clinical presentation of sclerodactyly and calcinosis cutis lesions on the left hand of a 66-year-old Caucasian female The inward curling of the patient's fingers, as well as the hard, whitish-yellow lesions (orange arrows) ranging in size from 0.5 x 0.5 cm to 1.5 x 1.0 cm, were present on both of the patient's hands (left > right). The black rectangle in the figure is concealing an identifying patient tattoo.

Upon inspection of vital signs and physical examination, it was noted that the patient was severely cachectic (BMI = 12.1 at a bodyweight of 30 kg) with a flat, rigid abdomen, and diffuse abdominal tenderness to palpation throughout all four quadrants. The patient also had very dry oral mucosal membranes without visible oropharyngeal lesions, erythema, or thrush. Cutaneous examination of her hands revealed inward curling of the fingers with hard, white-yellow lesions on multiple digits of both hands ranging in size from 0.5 x 0.5 cm to 1.5 x 1.0 cm (Figure 1). The remainder of the patient’s physical examination was unremarkable.

Labs taken in the emergency department were notable for severe hypokalemia and expected metabolic alkalosis. CT scan of the abdomen/pelvis endorsed an atrophic pancreas with potentially multi-cystic lobular disease; however, further imaging with MRI was recommended. The patient was admitted for a further workup to determine an etiology of her symptoms, as well as management of her failure to thrive and overall condition on presentation.

Immediate malignancy markers (CA-19-9, CA-125, carcinoembryonic antigen [CEA], and alpha-fetoprotein [AFP]) and an autoimmune panel consisting of anti-nuclear antibodies (ANA), anti-centromere, and anti-Scl-70 (among many others such as anti-smooth muscle, anti-mitochondrial, anti-histone, anti-Smith, etc.) antibodies were ordered due to high provider suspicion for malignancy or autoimmune disease. Per the recommendation of the abdomen/pelvis CT ordered in the emergency department, an MRI of the abdomen was also ordered.

**Figure 2:**
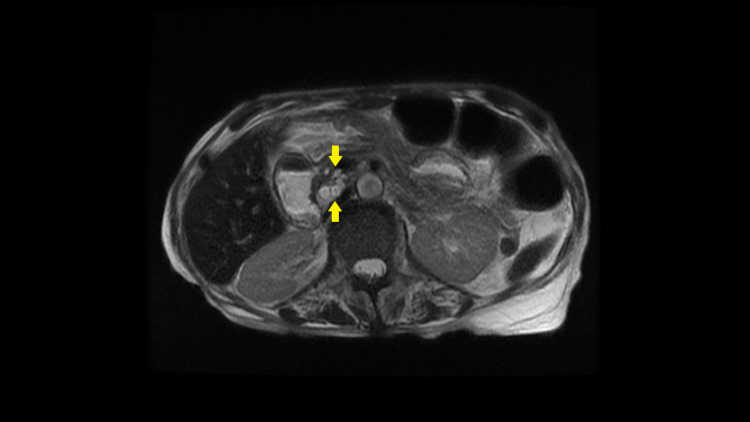
T1-weighted MRI scan of the abdomen with intravenous contrast, axial view, in a 66-year-old female presenting with four days of abdominal pain and nausea/vomiting Axial view of an MRI scan of the abdomen with intravenous contrast demonstrates a 1.7 x 1.4 x 2.0 cm cluster of multi-lobulated cystic lesions (yellow arrows), indicative of a side-branch intraductal papillary mucinous neoplasm (IPMN), abutting the medial/posterior aspect of the pancreatic head and neck.

Upon completion, an MRI of the patient’s abdomen yielded confirmation of pancreatic atrophy, as well as visualization of an IPMN due to higher resolution of the multi-cystic lobules in the pancreas (Figure 2). The reading radiologist recommended further outpatient follow-up with endoscopic ultrasound and potential biopsy by gastroenterology (the patient already had an upcoming appointment) together with repeat MRI in six months for surveillance of lesion progression.

The patient’s malignancy markers returned with a modestly elevated CA 19-9 of 68.5 (reference range was 2.0-37.0) and CA-125 of 42.4 (reference range was 1.5-35.0); her CEA and AFP were both within normal limits. Though these malignancy markers were elevated, per oncology consult, they were not at levels normally seen with pancreatic adenocarcinoma or ovarian cancer. The patient’s autoimmune panel returned positive for ANA and anti-centromere antibodies. When these lab values were taken into consideration with the patient’s presenting symptoms, past medical history, and physical examination findings, a diagnosis most consistent with lcSS was made. This was in addition to the patient’s newly diagnosed IPMN.

Throughout the course of her hospital stay, the patient underwent rigorous electrolyte repletion, fluid resuscitation, and nutritional supplementation (including pancreatic replacement therapy). These interventions yielded an approximate 15% increase in her bodyweight from admission. Her presenting symptoms were also managed via pain control plus anti-emetics and eventually resolved prior to discharge. Upon discharge, the patient was strongly recommended to maintain her gastroenterology outpatient appointment, as well as follow-up with an oncologist and rheumatologist, for her new diagnosed IPMN and lcSS.

## Discussion

Cancer has a common association with multiple autoimmune conditions. For example, patients with Sjogren syndrome are about 38 times more likely to develop lymphoma as compared to the general population. And other diseases such as dermatomyositis and polymyositis have shown a sixfold and twofold increase, respectively, in the development of cancer [4,5]. The association between malignancy and SS is one that is not clearly established; however, emerging data discussed in the ensuing paragraphs suggests increased cancer rates among SS patients. SS is broken down into both the subtype and the auto-antibody involved (Table 1). In lcSS, the presentation tends to involve the skin distal to the elbows and is typically associated with anti-centromere antibodies. In dcSS, the presentation tends to involve the skin proximal to the elbows and is typically associated with anti-Scl-70 antibodies [6].

**Table 1:** Features of limited cutaneous systemic sclerosis (lcSS) and diffuse cutaneous systemic sclerosis (dcSS) *CREST: Calcinosis Cutis, Raynaud Syndrome, Esophageal Dysmotility, Sclerodactyly, Telangiectasias

	lcSS	dcSS
Skin Involvement	Distal to elbows and knees [1]	Proximal to elbows and knees [1]
Raynaud Syndrome	Present [6]	Present [6]
Organ Involvement	Late-Onset: [1] Lung Esophageal	Early-Onset: [1] Lung Kidney Cardiac
Other Features	*CREST Variant [6]	Scleroderma Renal Crisis [6]
Auto-antibodies	Anti-Centromere [6]	Anti-Scl-70 [6]

Many epidemiological studies which include meta-analyses, cohort studies, and reviews, have suggested an increased risk of cancer in SS patients. The most common cancer found to be associated with SS is lung cancer [7]. Moreover, lung involvement is common with pulmonary hypertension and interstitial lung disease being the leading causes of death in all SS patients [8]. This suggests that the fibrosis and chronic inflammation that SS causes can lead to the development of malignancy. In addition to lung cancer, these studies also showed a link between SS and other cancers including breast cancer, hepatocellular carcinoma, esophageal cancer, lymphoma, and melanoma [7]. This diverse list of cancers illustrates the variability of SS, and it infers that the involved organ is at a higher risk of developing malignancy.

IPMNs are cystic tumors that produce mucin and involve the pancreatic ducts. The oncologic relevance of IPMNs stem from the fact that they are pre-cancerous lesions that have the ability to progress to pancreatic cancer, though most never undergo this transformation. This progression is due to mutations that cause an IPMN to become increasingly more dysplastic and eventually evolve into an invasive ductal carcinoma of the pancreas. IPMNs have been associated with smoking, diabetes, chronic pancreatitis, and a family history of pancreatic adenocarcinoma; however, no clear identifiable risk factors are well established. Additionally, there is limited data on the prevalence of IPMNs, but it is estimated that it is incidentally found in 3%-13% of the asymptomatic population. The usual diagnostic tool of choice is MRI or magnetic resonance cholangiopancreatography. Pancreatic biopsies are rarely performed in these patients due to the relatively low potential of an IPMN undergoing complete malignant transformation [9]. Also, the benefit of biopsying an IPMN to determine potential for malignant transformation must be balanced against the risk of inducing pancreatitis and its associated sequelae in this already vulnerable patient population.

The gastrointestinal system is affected in approximately 90% of SS patients. Nevertheless, the pancreatic effects of SS are poorly described. Some evidence indicates that SS affects the exocrine function of the pancreas. Typically the pancreatic involvement in SS is subclinical, but it may present with diarrhea or acute pancreatitis [10]. There is limited data showing a link between pancreatic neoplasms and SS. The only study we found to have statistical significance was a Korean study, which involved 112 SS patients. These patients were followed for six months and only one developed pancreatic cancer [11]. This study highlights the lack of clinical significance in the current data between pancreatic cancer and SS. In regards to pancreatic IPMNs, studies have shown an increased rate in patients with systemic autoimmune diseases [12]. However, no case reports or studies have discussed this association. As mentioned above, direct fibrosis of the pancreas may increase the risk of IPMNs and further development of pancreatic carcinoma. Another potential cause that may lead to an increased risk of pancreatic neoplasm could be SS overlap syndromes. In a retrospective study that included 220 patients with IPMNs, it showed that 11% of those patients had an autoimmune disease [12].

Auto-antibodies provide physicians with both diagnostic and prognostic information in regards to SS. Classically, anti-centromere and anti-Scl-70 antibodies are the main serologies used in SS patients, but many others are currently being investigated. These antibodies include anti-RNA Polymerase III, anti-U1-RNP, anti-U3-RNP, anti-Th/To, and anti-Pm/Scl antibodies [6]. Some evidence establishes that anti-RNA Polymerase III positivity has been linked to a higher incidence of cancer. The mechanism is not well understood, but some theories are that oncogenic mutations lead to the development of these auto-antibodies. Moreover, anti-RNA polymerase III may contribute to chronic inflammation and fibrosis, which leads to cancer development. In addition, patients that were serology negative (negative for anti-centromere, anti-Scl-70, and anti-RNA Polymerase III) may also be at an increased risk of developing cancer [13]. Auto-antibodies and the effects they have in patients with SS is a rapidly expanding field of research, and future studies are likely to provide more information on their association with cancer development.

Overall, this case report illustrates a patient with lcSS who presented with sclerodactyly, calcinosis cutis, and anti-centromere positivity found to have an IPMN. This case supports studies suggesting increased cancer risk in SS patients. It further demonstrates a correlation between IPMNs and systemic autoimmune diseases. Additionally, it adds value to patients with SS, which may require a more vigilant approach to cancer screening. This case also highlights a need for further research in regards to the pathogenesis of SS and cancer.

## Conclusions

SS is a chronic autoimmune condition that manifests with skin, subcutaneous, and internal organ involvement. It is a heterogenous disease with limited cutaneous and diffuse cutaneous phenotypes, and it can overlap with other autoimmune conditions. The association of SS and malignancy is one that is not clearly elucidated, and many studies have shown mixed results when it comes to cancer development. Additionally, the types of cancers that develop tend to be mixed, and the development of IPMN of the pancreas is not one that is commonly found in studies. Areas of interest for future studies can include SS auto-antibodies, phenotypes, and overlap syndromes. A better understanding of these components can highlight patients at higher risk for cancer and provide those patients with more individualized care.
